# CD47 Deficiency Protects Mice From Diet-induced Obesity and Improves Whole Body Glucose Tolerance and Insulin Sensitivity

**DOI:** 10.1038/srep08846

**Published:** 2015-03-09

**Authors:** Hasiyeti Maimaitiyiming, Heather Norman, Qi Zhou, Shuxia Wang

**Affiliations:** 1 Department of Pharmacology and Nutritional Sciences, University of Kentucky, Lexington, KY 40536; Lexington Veterans Affairs Medical Center, Lexington, KY

## Abstract

CD47 is a transmembrane protein with several functions including self-recognition, immune cell communication, and cell signaling. Although it has been extensively studied in cancer and ischemia, CD47 function in obesity has never been explored. In this study, we utilized CD47 deficient mice in a high-fat diet induced obesity model to study for the first time whether CD47 plays a role in the development of obesity and metabolic complications. Male CD47 deficient and wild type (WT) control mice were fed with either low fat (LF) or high fat (HF) diets for 16 weeks. Interestingly, we found that CD47 deficient mice were protected from HF diet-induced obesity displaying decreased weight gain and reduced adiposity. This led to decreased MCP1/CCR2 dependent macrophage infiltration into adipose tissue and reduced inflammation, resulting in improved glucose tolerance and insulin sensitivity. In addition, CD47 deficiency stimulated the expression of UCP1 and carnitine palmitoyltransferase 1b (CPT1b) levels in brown adipose tissue, leading to increased lipid utilization and heat production. This contributes to the increased energy utilization and reduced adiposity observed in these mice. Taken together, these data revealed a novel role for CD47 in the development of obesity and its related metabolic complications.

Obesity and its-associated insulin resistance is rampant within the United States and other developed nations. Previous studies from our lab and others suggest that thrombospondin 1 (TSP1) plays an important role in obesity-associated chronic inflammation and insulin resistance (IR)^1^,^2^,^3^,^4^. We demonstrated that TSP1 deficiency did not affect the development of high-fat diet induced obesity. However, TSP1 deficiency reduced macrophage accumulation in adipose tissue and protected against obesity related inflammation and insulin resistance^3^. These data suggest that TSP1 plays an important role in regulating macrophage function and mediating obesity-induced inflammation and insulin resistance. However, the mechanisms of the pro-inflammatory effect elicited by TSP1 under obese conditions remain to be determined.

TSP1, a 420–450 kDa homotrimer, is a multifunctional matricellular protein composed of several domains that can interact with different cell surface receptors^5^,^6^,^7^,^8^,^9^,^10^,^11^,^12^,^13^,^14^,^15^,^16^. CD47 is one of the TSP1 receptor and a trans-membrane glycoprotein cell receptor that belongs to the immunoglobulin superfamily^17^. CD47 expression can be seen in various tissues and cell types throughout the body, ranging from microglia to red blood cells^18^,^19^. Wide expression suggests that CD47 is active or necessary in several different cellular pathways including immunity and self-recognition, inflammation, cellular adhesion, stress response, cell survival, and vascular function^20^,^21^,^22^,^23^,^24^. It has been shown that TSP1-CD47 interaction inhibits NO/cGMP/PKG signaling in vascular smooth muscle cells and plays a role in vasoconstriction and inflammation^22^,^25^,^26^,^27^. However, it is unknown whether the pro-inflammatory effect elicited by TSP1 under obese conditions is mediated by CD47.

In the current study, we determined whether TSP1 promotes obesity-associated inflammation and insulin resistance is through interaction with its receptor-CD47. We utilized CD47 deficient and WT mice in a diet-induced obesity paradigm. CD47 deficient mice challenged with a HF diet had several protective phenotypes as previously observed in TSP1 deficient mice including decreased obesity-associated inflammation and improved glucose tolerance and insulin sensitivity^3^. However, one different phenotype was observed between CD47 deficient mice and our previous HF-fed TSP1 deficient mice^3^, which was the significant changes in body weight. Current studies have shown that CD47 deficiency protected mice from HF diet induced obesity; while TSP1 deficiency had no effect on diet-induced obesity after 16 weeks of HF feeding. Moreover, CD47 deficiency increased energy expenditure, heat production and core body temperature partially relating to brown adipose tissue and/or skeletal muscle functional changes. Together, data from this study revealed a novel role for CD47 in regulation of energy homeostasis and the development of obesity, suggesting that CD47 may serve as a potential therapeutic target to combat obesity and metabolic complications.

## Results

### CD47 deficiency protects mice from diet-induced obesity

**Figure 1:**
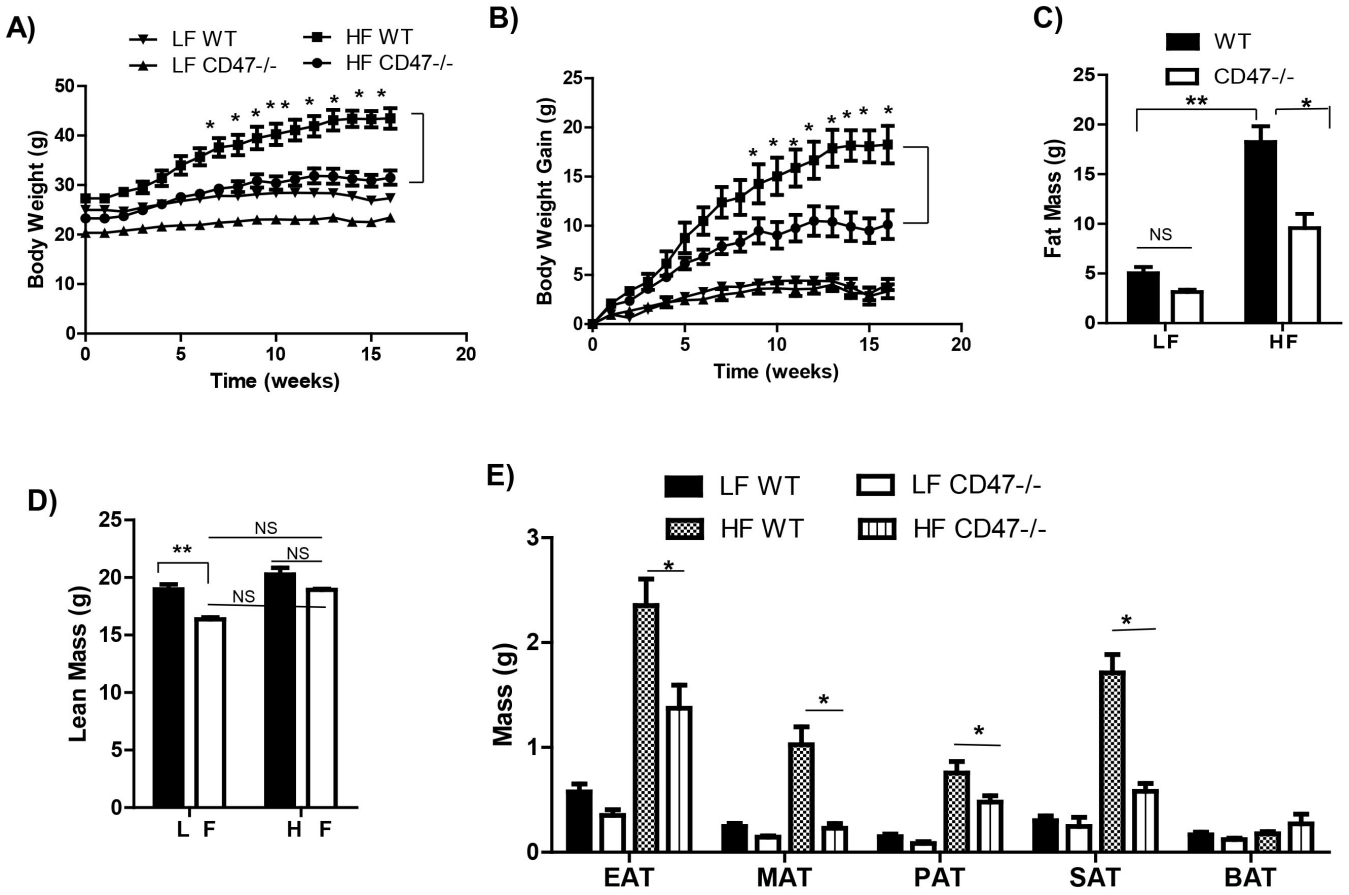
CD47 deficient mice were protected from high fat diet-induced obesity. Eight week old male CD47 deficient mice and wild type C57BL6 controls were fed with low fat (LF) or high fat (HF) diet for 16 weeks. Weekly body weight (A) and body weight gain (B) were shown. Fat (C) and lean mass (D) of mice were measured by EchoMRI. (E) Absolute weight of white and brown adipose tissues was measured immediately following sacrifice. Data are presented as mean ± SE (n = 7 mice/group), *p < 0.05, **p < 0.01. EAT: epididymal adipose tissue; MAT: mesenteric adipose tissue; PAT: perirenal adipose tissue; SAT: subcutaneous adipose tissue; BAT: brown adipose tissue.

To assess the metabolic role of CD47 in mice, CD47 deficient mice and WT controls were challenged with either a low fat (LF, 10% kcal from fat) or high fat (HF, 60% kcal from fat) diet for 16 weeks. Under LF diet, although CD 47 deficient mice had a trend of decrease in body weight as compared to WT mice, no significant changes were observed throughout the study (Fig. 1A). When challenged with HF diet, CD47 deficient mice exhibited significantly reduced body weight starting from 7 weeks' feeding till the end of the study. These mice gained less weight than HF-fed WT mice (Fig. 1B). At the end of study, body composition was determined in mice by using EchoMRI. There was significantly reduced fat mass in HF-fed CD47 deficient mice as compared to HF-fed WT mice (Fig. 1C), which was in agreement with the absolute weight of different adipose tissue depots (Fig. 1E). Lean mass was comparable in HF-fed CD47 deficient mice and WT mice (Fig. 1D). Consistent with the decreased adiposity, HF-fed CD47 deficient mice had reduced leptin levels (ng/ml, WT HF: 14.4 ± 4.89 vs. CD47-/- HF: 6.83 ± 1.09, p < 0.05). Moreover, plasma total cholesterol (TC) and free fatty acid (FFA) levels were significantly reduced in HF-fed CD47 deficient mice (TC (mg/dl), WT HF: 115.08 ± 7.25 vs. CD47-/- HF: 80.04 ± 7.87, p < 0.01; FFA (mEq/L), WT HF: 0.34 ± 0.01 vs. CD47-/- HF: 0.28 ± 0.02, p < 0.05). Together, these data suggests that CD47 deficiency protects mice from diet-induced obesity.

### CD47 deficient mice on the HF diet showed reduced systemic and adipose tissue inflammation

**Figure 2:**
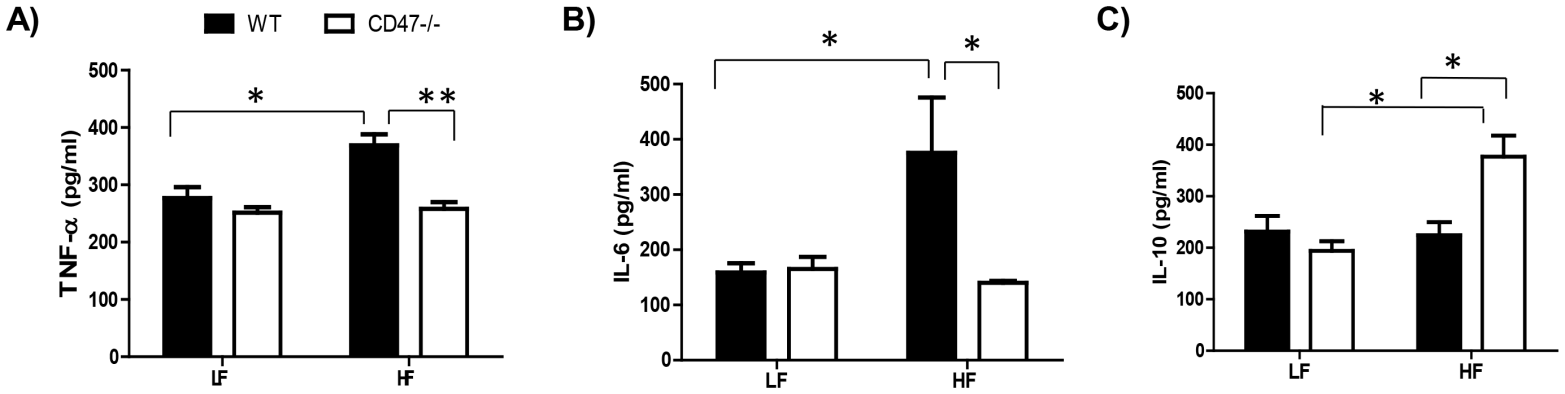
HF-fed CD47 deficient mice displayed reduced systemic inflammation compared to HF-fed wild type controls. Plasma TNF-α (A), IL-6 (B), and IL-10 (C) levels were measured by ELISA as described in Methods. Data are presented as mean ± SE (n = 7 mice/group), *P < 0.05 and ** P < 0.01.

**Figure 3:**
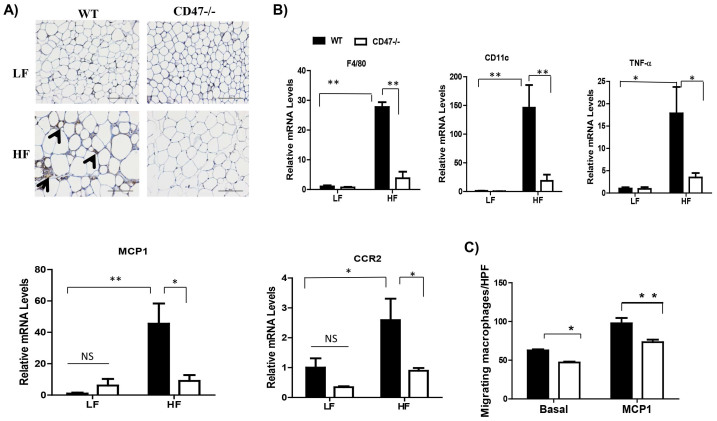
HF-fed CD47 deficient mice had decreased adipose tissue macrophage infiltration and inflammation. (A) Macrophage accumulation in epididymal adipose tissue was determined by anti-F4/80 staining. The positive staining showed brown color and indicated by arrow head. The representative images are shown. Scale bars represent 100 μm. (B) Expression of pro-inflammatory cytokines in epididymal adipose tissue was determined by real-time PCR and normalized to 18S RNA. (C) Bone marrow cells from HF-fed WT and CD47 deficient mice were isolated and differentiated into macrophages. Migration of these cells were measured basally and upon MCP1 (50 ng/ml) stimulation using modified Boyden Microchemotaxis Chamber. Data are presented as mean ± SE (n = 7 mice/group), *p < 0.05 and **p < 0.01.

Systemic as well as adipose tissue inflammation were determined in four groups of mice. As shown in Fig. 2A,B, HF-fed CD47 deficient mice had significant reduction in plasma TNF-α and IL-6 levels. In addition to the reduced plasma pro-inflammatory cytokines, plasma anti-inflammatory cytokine-IL-10 levels were significantly increased in HF-fed CD47 deficient mice as compared to HF-fed WT mice or to LF-fed CD47 deficient mice (Fig. 2C), suggesting that the interaction between diet composition and genotype contributes to the IL-10 secretion. In addition to systemic inflammation, adipose tissue inflammation status was determined. Visceral adipose tissue has been suggested to be the primary source of cytokine and adipokine release within obesity-associated inflammation^28^. Moreover, increased accumulation of adipose tissue macrophages is a significant contributor to obesity- induced chronic inflammation^29^,^30^,^31^. Therefore macrophage infiltration into adipose tissue was determined by immunohistochemical staining for macrophage marker-F4/80. As shown in Fig. 3A, HF-fed WT controls had robust positive staining of F4/80 and crown-like structures, yet HF-fed CD47 deficient mice had minimal positive staining, suggesting a decreased presence of macrophages. We confirmed this staining result with qPCR and demonstrated that HF-fed WT controls had a significant increase in F4/80 expression in adipose tissue, which was reduced in HF-fed CD47 deficient mice (Fig. 3B). Moreover, CD11c and TNF-α levels were increased in adipose tissue from HF-fed WT mice, but decreased in HF-fed CD47deficient mice (Fig. 3B). Together, these data indicate that HF-fed CD47 deficient mice had reduced macrophage infiltration into adipose tissue and decreased systemic and adipose tissue inflammation.

To determine the mechanism of reduced macrophage infiltration in adipose tissue from HF-fed CD47 deficient mice, we examined MCP1 and CCR2 levels. MCP1, an inflammatory chemokine responsible for monocyte migration, and its dominant receptor CCR2 are suggested to be responsible for a significant amount of monocyte infiltration into inflamed adipose tissue^29^. We found that HF-fed CD47 deficient mice demonstrated a reduction in MCP1 and CCR2 levels in adipose tissue (Fig. 3B), which might be due to the reduced adiposity in these mice. In addition, this result could explain the significant decrease in macrophage infiltration in adipose tissue from CD47 deficient mice, which was supported by an *in vitro* migration studies using isolated macrophages from HF fed WT and CD47 deficient mice. We found that WT macrophages had increased MCP1 stimulated migration; while macrophages from CD47 deficient mice showed reduced migration in response to MCP1 (Fig. 3C). Together, these data suggest that CD47 regulates diet-induced adipose tissue macrophage infiltration and inflammation through a MCP1/CCR2 dependent pathway.

### CD47 deficient mice on the HF diet showed improved whole body glucose homeostasis

**Figure 4:**
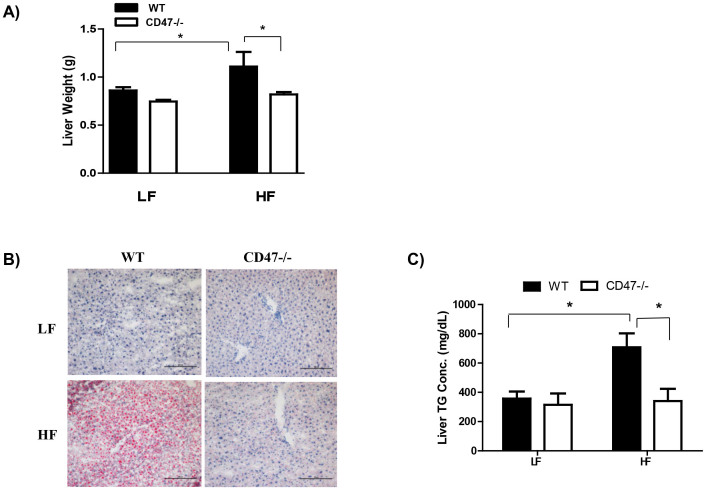
CD47 deficiency prevents lipid accumulation in liver after HF diet feeding. Lipid accumulation in liver from four groups of mice was determined by measuring liver weight (A), staining liver sections by Oil-red-O (B), and quantification of triglyceride from liver extracts (C). The representative images of Oil-red-O staining are shown. Scale bars represent 100 μm. Data are presented as mean ± SE (n = 7 mice/group), *P < 0.05.

The liver morphology was determined in the current study. We found that CD47 deficiency protected mice from hepatosteatosis when fed with HF diet, which was demonstrated by reduced liver weight, oil red-O staining in liver sections and liver triglyceride levels (Fig. 4).

**Figure 5:**
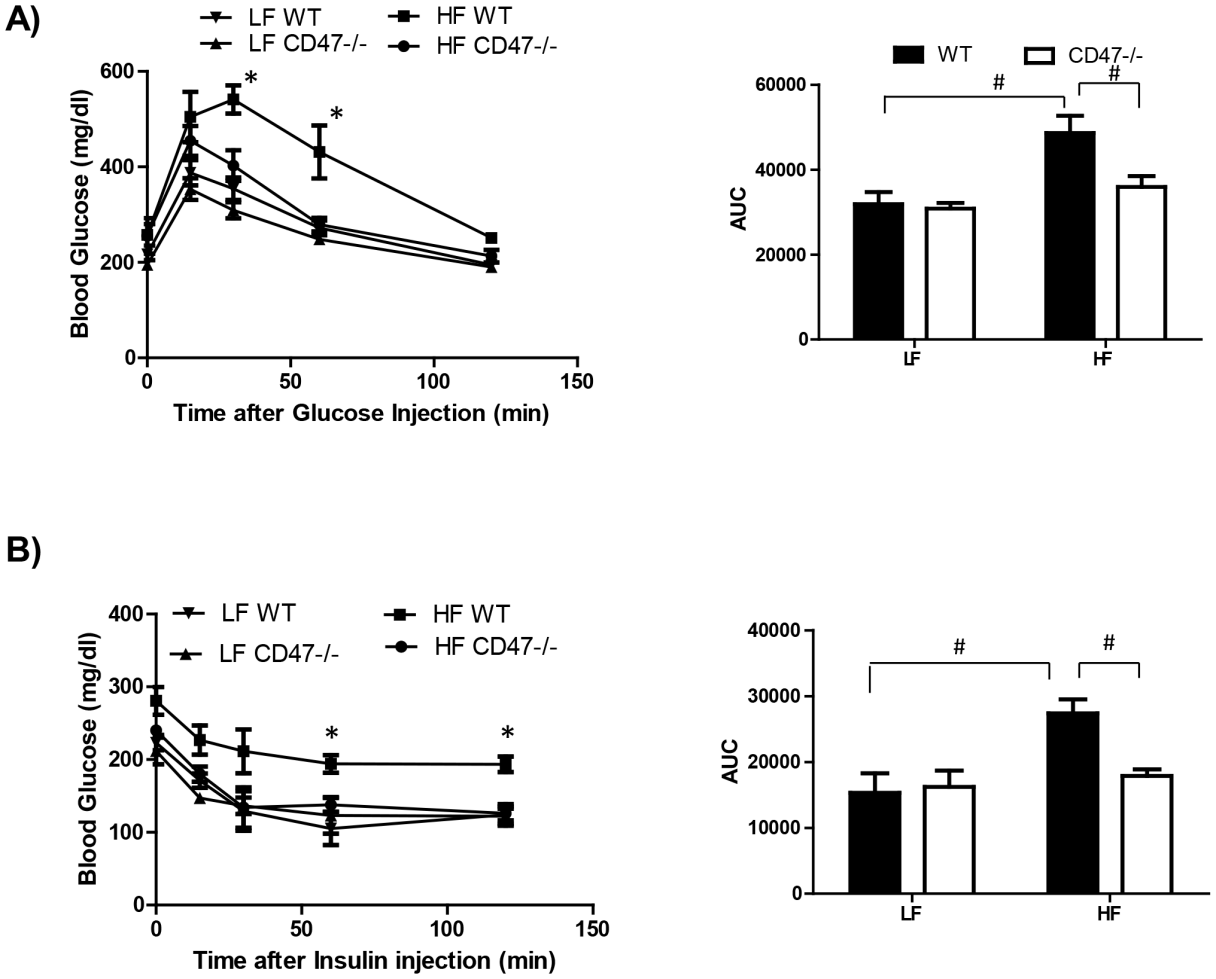
HF-fed CD47 deficient mice had improved glucose tolerance and insulin sensitivity. Intraperitoneal glucose tolerance test (A) and insulin sensitivity test (B) were performed in male CD47 deficient and littermate control mice after 15 weeks of HF or LF feeding. Data are presented as mean ± SE (n = 7 mice/group), *P < 0.05 vs. HF CD47-/-; #P < 0.05; AUC: area under the curve.

To determine the whole body glucose homeostasis, we performed glucose tolerance (GTT) and insulin sensitivity tests (ITT) in WT and CD47 deficient mice. As shown in Fig. 5, glucose tolerance and insulin sensitivity were significantly improved in HF-fed CD47-/- mice as compared to HF-fed WT mice, suggesting that CD47 deficiency protects mice from diet-induced glucose intolerance and insulin resistance.

### Energy metabolism in WT or CD47 deficient mice under either LF or HF feeding conditions

**Figure 6:**
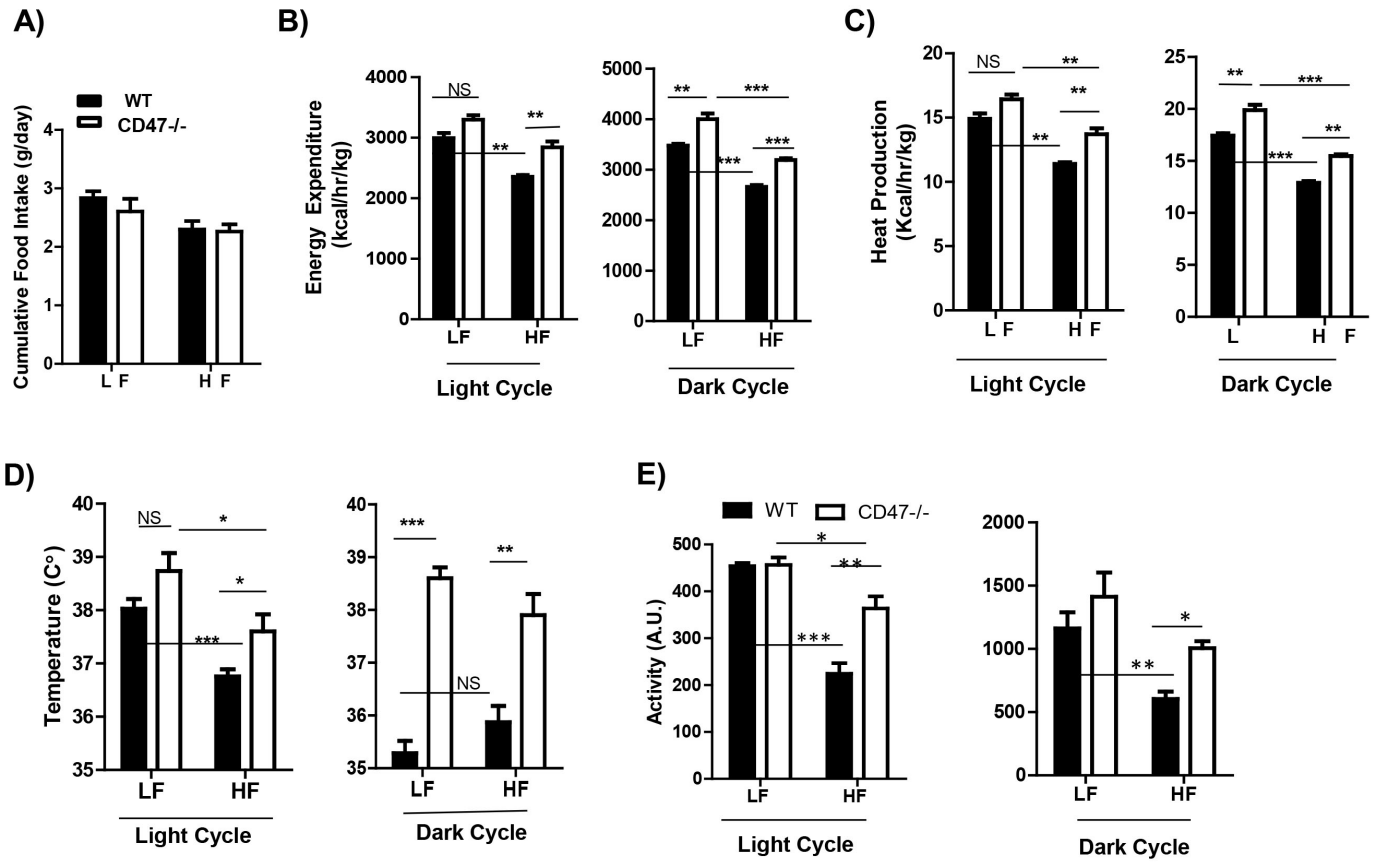
Energy metabolism in WT and CD47 deficient mice under either LF or HF feeding conditions. Eight week old male CD47 deficient mice and wild type littermate controls were fed with low fat (LF) or high fat (HF) diet for 16 weeks and housed in TSE chambers for indirect calorimetric analysis. (A) Daily food intake was measured for 6 consecutive days. (B) Energy expenditure was normalized to total body mass during both the light and dark cycles. (C) Heat production, (D) Core body temperature, and (E) Activity were shown during both the light and dark cycles. Data are presented as mean ± SE (n = 4 mice/group), *P < 0.05; **p < 0.01; ***P < 0.001.

We have shown that CD47 deficiency protects mice from diet-induced obesity. To further elucidate its mechanism, we examined the energy balance in both WT and CD47 deficient mice. Daily cumulative food consumption was measured and there was no significant difference between either genotype or diet type (Fig. 6A). However, CD47 deficient mice displayed elevated energy expenditure (normalized to total body mass), heat production, core body temperature, or total activity compared to WT mice under HF feeding conditions in either light or dark cycle (Fig. 6B–E). Together, these data suggest that the protection against HF diet induced weight gain and fat gain in CD47 deficient mice might be due to increased energy utilization.

### Metabolic gene expression in skeletal muscle from LF or HF fed WT and CD47 deficient mice

**Figure 7:**
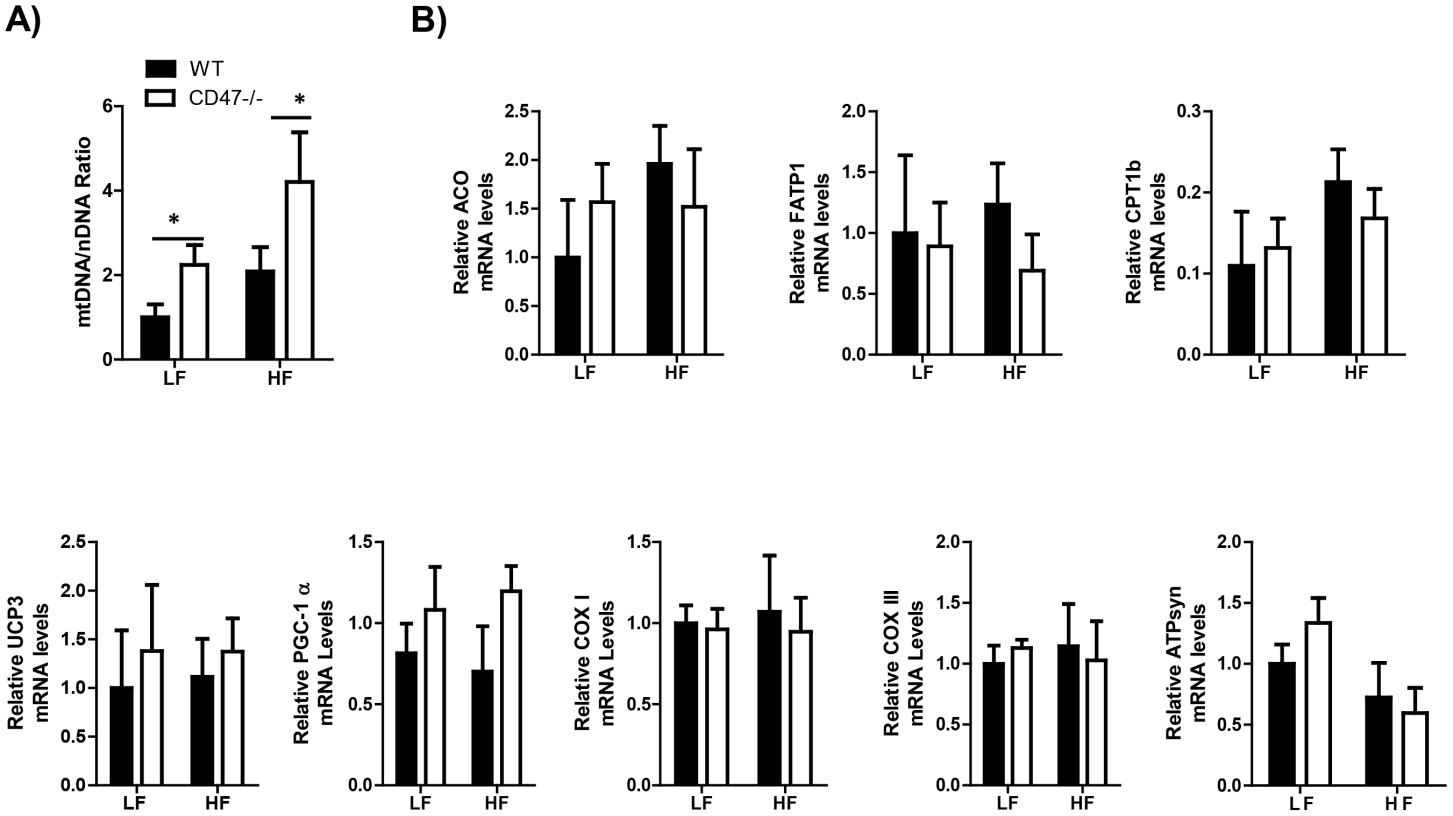
Metabolic gene expression in skeletal muscle from LF or HF feeding WT or CD47 deficient mice. (A) Mitochondria DNA copy number in skeletal mice from four groups of mice and (B) expression of metabolic genes including acylcoA Oxidase (ACO), Fatty acid transporter protein (FATP1), Carnitine palmitoyltransferase 1b (CPT1b), UCP3, PGC-1α, COX I, COX III and ATPsyn by real-time PCR. Data are presented as mean ± SE (n = 6–7 mice/group), *P < 0.05.

To further determine the mechanism of increased energy utilization in HF-Fed CD47 deficient mice, first, we analyzed the expression of multiple genes in skeletal muscle that relate to mitochondria function and fuel utilization. The rationale for this analysis was based on the previous report showing that skeletal muscle from CD47 deficient mice had greater number of mitochondria and improved function^32^, which suggested a possible contribution of skeletal muscle to the metabolic phenotype observed in the current study. Therefore, mitochondria DNA copy number and expression of a series genes relating to mitochondria oxidative function and fatty acid catabolism were analyzed. The results showed that mitochondria DNA copy number was significantly increased in CD47 deficient mice compared to WT mice under either LF or HF feeding conditions (Fig. 7A). However, expression levels of genes relating to mitochondria oxidative function and fatty acid catabolism were comparable in CD47 deficient mice compared to WT mice under either LF or HF feeding conditions (Fig. 7B), suggesting that skeletal muscle functional change might be not the major contribution to the increased energy utilization phenotype in HF-fed CD47 deficient mice.

### Morphology and metabolic gene expression in brown adipose tissue from LF or HF fed WT and CD47 deficient mice

**Figure 8:**
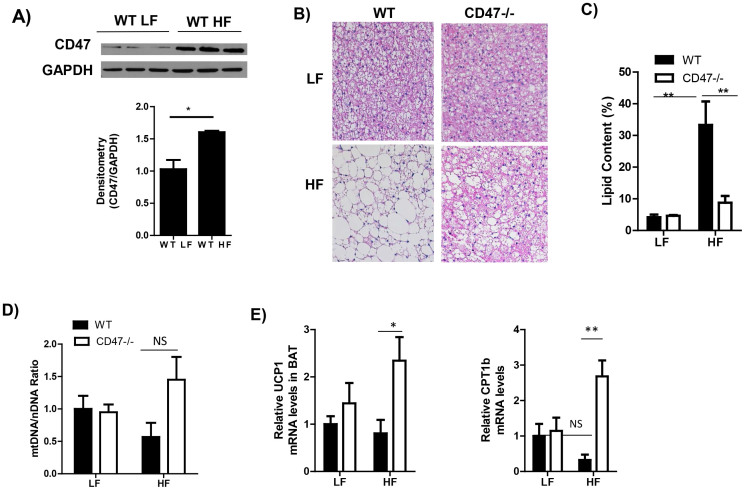
Morphology and metabolic gene expression in brown adipose tissue from LF or HF feeding WT or CD47 deficient mice. (A) CD47 protein levels in brown adipose tissue from LF or HF fed WT mice by immunoblotting (Cropped blots were used); (B) Representative images of HE staining of brown adipose tissue from LF or HF fed CD47 deficient and WT mice. Images were obtained at x20. Scale bars represent 100 μm; (C) Percentage lipid content in brown adipose tissue sections, as quantified using image analysis software. (D) Mitochondria DNA copy number by PCR and (E) Expression of metabolic genes by real-time PCR. Data are presented as mean ± SE (n = 6–7 mice/group), *P < 0.05 and **p < 0.01.

Recently, brown adipose tissue (BAT) emerged as an important player in energy metabolism^33^. However, whether CD47 regulates BAT function and contributes to diet-induced obesity is unknown. First, we found that diet-induced obesity significantly up-regulated CD47 protein levels in BAT in wild type mice (Fig. 8A). Although interscapular BAT weight was not different between WT and CD47-/- mice under either LF or HF feeding conditions (Fig. 1E), histology showed a decrease in intracellular lipid droplet size in BAT of HF-fed CD47 deficient mice compared to HF-fed WT mice, reflected by a decrease in relative lipid area (Fig. 8B, C). Moreover, we analyzed mitochondria DNA copy number and a series of genes relating to mitochondria oxidative function and fatty acid catabolism. As shown in Fig. 8D, no significant changes in mitochondria DNA copy number in BAT was observed in CD47-/- mice compared to WT mice, suggesting that CD47 does not affect mitochondria biogenesis in BAT. In addition, mRNA levels of UCP1 and carnitine palmitoyltransferase 1B (CPT1b) in BAT were significantly increased in HF-fed CD47 deficient mice (Fig. 8E). BAT expends a large amount of energy through mitochondria β-oxidation and by uncoupling of the mitochondria proton gradient from ATP production. This uncoupling results in heat production or thermogenesis, accomplished by UCP1 located in the inner mitochondria membrane^34^,^35^. CPT1, located on the outer mitochondrial membrane, is the first and rate-limiting step for fatty acid transporting into the mitochondria for utilization^36^. Therefore, this data suggests that CD47 deficiency-mediated increased CPT1b expression in BAT may lead to increased fatty acid uptake in mitochondria and then activation of UCP1, resulting in increased uncoupling and heat production (Fig. 6C,D).

### cGMP/PKG signaling in WT or CD47 deficient mice under either LF or HF feeding conditions

**Figure 9:**
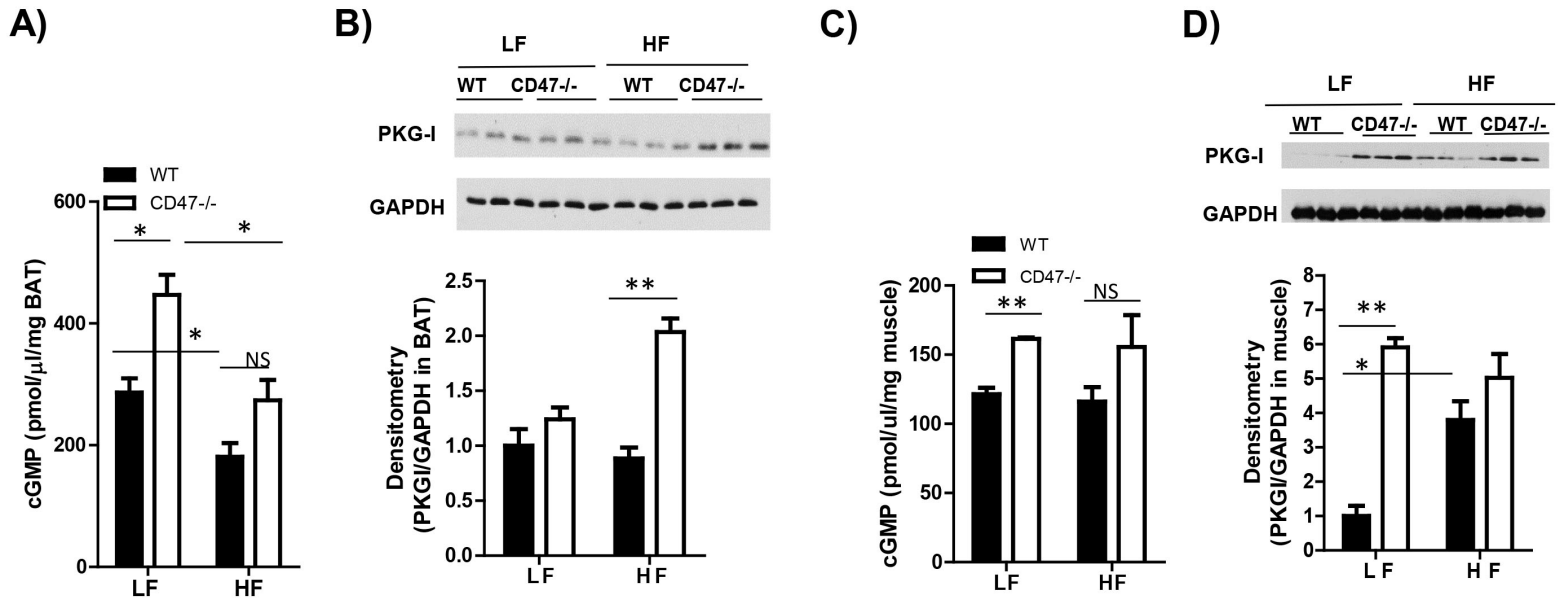
cGMP or PKG signaling in brown adipose tissue and skeletal muscle from LF or HF feeding WT or CD47 deficient mice. (A) cGMP levels in brown adipose tissue from LF or HF fed WT mice by direct immunoassay; (B) PKG-I protein levels in brown adipose tissue from LF or HF fed WT mice by immunoblotting (Cropped blots were used); (C) cGMP levels in skeletal muscle from LF or HF fed WT mice by direct immunoassay; (D) PKG-I protein levels in skeletal muscle from LF or HF fed WT mice by immunoblotting (Cropped blots were used). Data are presented as mean ± SE (n = 6 mice/group), *P < 0.05 and **p < 0.01.

Studies have shown that CD47 activation via TSP1 can disrupt NO/cGMP/PKG signaling in vascular cells^23^,^37^,^38^. Therefore, we determined whether cGMP/PKG signaling in brown adipose tissue (BAT) or skeletal muscle was changed in CD47-/- mice under either LF or HF feeding conditions. As shown in Fig. 9A, under LF feeding conditions, cGMP levels in BAT was increased in CD47-/- mice compared to WT mice. HF diet feeding significantly reduced BAT cGMP levels in WT or in CD47-/- mice compared to their LF controls. There was a trend of increase in BAT cGMP levels in HF-fed CD47-/- mice compared to HF-fed WT mice. For PKG-I protein levels in BAT, under LF feeding conditions, no difference was found between WT and CD47-/- mice. However, under HF feeding conditions, BAT PKG-I protein levels were significantly increased in CD47-/- mice compared to WT mice (Fig. 9B).

In addition to brow fat, cGMP/PKG signaling in skeletal muscle was analyzed. We found that cGMP levels or PKG-I protein levels were increased in LF-fed CD47-/- mice compared to LF-fed WT mice (Fig. 9C, D). However, under HF feeding conditions, there was no difference in cGMP or PKG-I levels between WT and CD47-/- mice. Together, these data suggest that cGMP/PKG signaling was differentially regulated by CD47 in BAT and skeletal muscle.

## Discussion

CD47 is a transmembrane protein with several functions including self-recognition, immune cell communication, and cell signaling^18^,^19^. Although it has been extensively studied in cancer and ischemia^39^,^40^,^41^,^42^, CD47 function in obesity has never been explored. In this study, we utilized CD47 deficient mice in a high-fat diet induced obesity model to study for the first time whether CD47 plays a role in diet-induced obesity and its associated metabolic complications. We found that CD47 deficient mice were protected from HF diet-induced obesity displaying decreased weight gain and reduced adiposity. This led to decreased MCP1/CCR2 dependent macrophage infiltration into adipose tissue and reduced inflammation, resulting in improved glucose tolerance and insulin sensitivity. In addition, CD47 deficiency stimulated the expression of UCP1 and CPT1b levels in brown adipose tissue, leading to increased lipid utilization and heat production and contributing to increased energy utilization and reduced adiposity in these mice. These data suggest a novel role for CD47 in regulation of brown adipose tissue function and its contribution to the development of obesity and dyslipidemia.

CD47 is a receptor for the matricellular protein-thrombospondin 1 (TSP1). Previous studies from our lab and others suggest that TSP1 plays a role in obesity-associated chronic inflammation and insulin resistance (IR)^1^,^2^,^3^,^4^. Both TSP1 and CD47 expression in adipose tissue was up-regulated under obese conditions^2^,^4^, suggesting that CD47 may mediate TSP1's effect on diet-induced obesity and obesity-associated complications. By feeding CD47 deficient mice with the same diet (10% (LF) and 60% fat (HF) diet) for same periods (16 weeks) as we did before for TSP1 deficient mice^3^, we found that most of the phenotypes observed in the TSP1 deficient mice undergoing HF feeding were replicated in CD47 deficient mice including reduced macrophage infiltration into adipose tissue, reduced inflammation, and improved glucose tolerance and insulin sensitivity. However, one different phenotype was observed between CD47 deficient mice and our previous HF-fed TSP1 deficient mice^3^, which was the significant changes in body weight. Current studies showed that CD47 deficiency protected mice from HF diet induced obesity; while TSP1 deficiency had no effect on diet-induced obesity after 16 weeks of HF feeding. This different phenotype suggests that TSP1-CD47 ligation may not be involved in regulation of energy homeostasis under HF feeding conditions. The novel CD47 ligands and their interaction on energy balance and the development of obesity warrant further investigation.

It has been shown that CD47 activation via TSP1 can disrupt NO-cGMP signaling and that decreased NO-cGMP pathway contributes to vascular inflammation as well as adipose tissue inflammation, resulting in insulin resistance^37^,^38^. In agreement with these observations, HF-fed CD47 deficient mice showed decreased levels of circulating proinflammatory cytokines as well as reduced inflammation in adipose tissue. They also had improved glucose tolerance and insulin sensitivity. Moreover, with reduced adiposity in HF-fed CD47 deficient mice, these mice exhibited reduced expression of MCP1 and CCR2 in adipose tissue. This was associated with reduced macrophage infiltration into adipose tissue in HF-fed CD47 deficient mice. Further *in vitro* analysis demonstrated that CD47 deficient macrophages had significantly decreased migration compared to WT cells upon stimulation with MCP1. The importance of MCP1 and CCR2 in adipose tissue macrophage recruitment and their contribution to insulin resistance has been demonstrated by numerous studies^43^,^44^,^45^,^46^,^47^,^48^. Thus, data from our study suggest that the effect of CD47 on macrophage infiltration into adipose tissue and the development of chronic inflammation under obesity conditions is MCP1/CCR2 dependent.

In this study, we found that CD47 deficiency protected mice from HF diet induced obesity, which was associated with increased energy utilization. To determine the mechanisms of increased metabolic rate in HF-fed CD47 deficient mice, we analyzed skeletal muscle function since a previous report showed that skeletal muscle from CD47 deficient mice had greater number of mitochondria and improved skeletal muscle function^32^. Although we saw increased mitochondria number in skeletal muscle from HF-fed CD47 deficient mice compared to HF-fed WT mice, the expression of genes relating to mitochondria oxidative function or fatty acid catabolism in skeletal muscle were comparable between WT and CD47 deficient mice (Fig. 7). These data suggest that skeletal muscle functional change might be not the major contribution to the increased energy expenditure phenotype in HF-fed CD47 deficient mice.

The contribution of adipose tissue function to the increased metabolic rate in HF-fed CD47 deficient mice was also analyzed. The rationale for such study is based on previous reports showing that increased cGMP/PKG signaling pathway stimulates brown adipocyte differentiation^49^, promotes healthy expansion and browning of white adipose tissue^50^, stimulates white adipose tissue lipolysis and cold induced brown fat thermogenesis^51^. In the current study, we found that obesity stimulated CD47 expression in brown adipose tissue (BAT). BAT is highly vascularized, highly innervated by the sympathetic nervous system, and is densely packed with mitochondria. BAT expends a large amount of energy through mitochondria β-oxidation and by uncoupling of the mitochondria proton gradient from ATP production. This uncoupling results in heat production or thermogenesis, accomplished by UCP1 located in the inner mitochondria membrane^34^,^35^. In the current study, we found that brown fat mass and mitochondria DNA content was similar between WT and CD47 deficient mice. UCP1 expression was not found in white fat from either WT or CD 47 deficient mice under either LF or HF feeding conditions (data not shown), suggesting there was no browning of white fat. Importantly, loss of CD47 caused significantly increased UCP1 expression in brown fat under HF feeding conditions. Consistently, we found that core body temperature was elevated in HF-fed CD47 deficient mice, indicating the increased thermogenesis. It is known that fatty acids are an important fuel for thermogenesis. Lipolysis releases fatty acids that can be used for mitochondria oxidation and thermogenesis. Previously, we found that activation of PKG signaling stimulates lipolysis in adipose tissue^51^. In the current study, we demonstrated that cGMP and/or PKG signaling was up-regulated in brown fat from CD47 deficient mice. Moreover, the lipid accumulation in the brown fat was significantly reduced in HF-fed CD47 deficient mice. Based on these data, we speculate that CD47 deficiency stimulates lipolysis in brown fat via activation of cGMP/PKG pathway. In addition, we found that the carnitine palmitoyltransferase 1 (CPT1) was up-regulated in HF-fed CD47 deficient mice compared to HF-fed WT mice. CPT1, located on the outer mitochondrial membrane, is the first and rate-limiting step for fatty acid transporting into the mitochondria for utilization^36^. Therefore, CD47 deficiency may increase fatty acid translocation into mitochondria by fatty acid transporter CPT1 and then lead to the activation of UCP1 in brown fat and increased heat production. Our data suggest that CD47 mediated regulation of brown fat function contributes to the metabolic phonotype observed in the current study. However, to definitively demonstrate the effect of brown fat cell derived CD47 on energy metabolism in diet-induced obesity, the tissue specific CD47 deficient mice are required in future studies.

In summary, for the first time, our studies demonstrate an important role for CD47 in regulating energy balance and the development of obesity and its metabolic complications. CD47 deficiency protects mice from HF diet-induced obesity through stimulation of energy expenditure and heat production. In addition, CD47 deficiency reduces obesity- associated metabolic complications including decreased systemic and adipose tissue inflammation and hepatosteatosis, and improved glucose tolerance and insulin sensitivity. The results from this study suggest that CD47 may serve as a therapeutic target of obesity and its related comorbidities.

## Methods

### Animal experimental protocol

All experiments were performed on eight week-old male CD47-/- mice (C57BL6/J background from Jackson Laboratories) and same sex and age-matched C57BL6/J controls. Mice were given a high fat (HF) (60% kcal from fat; D12492, Research Diets, Inc, NJ) or low fat (LF) diet (10% kcal from fat; D12450B; Research Diets, Inc, NJ) for 16 weeks with standard laboratory water. Each group contained 7 mice. Body weight was measured weekly at the same time. Temperature transponders (Implantable Programmable Temperature Transponder 300; BioMedic Data Systems, Seaford, DE) were subcutaneously implanted into mice at the week 8 time point during diet challenge. The wireless reader system was utilized to measure core body temperatures in both the light and dark cycles. At the end of the study, mice were sacrificed. Blood was collected and adipose tissue depots and muscle were harvested for various analyses. All experiments involving mice conformed to the National Institutes of Health Guide for the Care and Use of Laboratory Animals and were approved by the University of Kentucky Institutional Animal Care and Use Committee.

### Indirect calorimetry and body composition

Two weeks prior to the end of study, mice were placed in TSE LabMaster chambers (TSE systems) individually for 5 days for measurement of food intake, water intake and indirect calorimetry. Body composition including lean and fat mass was measured by EchoMRI (Echo Medical System) basally and after 16 weeks of HF/LF feeding.

### Plasma parameters analysis

Plasma insulin, IL-6, TNFα, IL-10, leptin, and MCP1 (eBioscience, USA) were measured by ELISA. Plasma and liver triacylglycerol (TG), non-esterified fatty acids (NEFA) and total cholesterol (Wako Chemicals, Richmond, USA) levels were measured enzymatically.

### Intraperitoneal glucose (GTT) and insulin (ITT) tolerance test

Glucose tolerance and insulin tolerance were analyzed basally and after 15-weeks of HF or LF feeding. Mice were fasted 6 hours before intraperitoneal injections of glucose (1 g/kg body weight) or insulin (0.5 unit/kg body weight; Novolin R, Novo Nordisk Inc.). Blood glucose concentrations were measured using a glucometer at 0, 15, 30, 60, and 120 minutes post injection.

### Real-time quantitative PCR

Total RNA from frozen adipose tissue and skeletal muscle were extracted using RNeasy Mini Kit (Qiagen, USA). RNA was reverse transcribed to cDNA by High Capacity cDNA Reverse Transcription Kit (Invitrogen, Carlsband, CA). Real-time quantitative PCR was performed on a MyiQ Real-time PCR Thermal Cycler (Bio-Rad) with SYBR Green PCR Master Kit (Qiagen, Valencia, CA). Relative mRNA expression was calculated using the MyiQ system software as previous reported^3^ and normalized to 18 s RNA levels. All primer sequences utilized in this study are found in supplementary Table 1.

### Mitochondrial DNA copy number

DNA was extracted from skeletal muscle or brown adipose tissue by using QIAamp DNA mini kit (Qiagen). The relative mitochondria DNA (mtDNA) copy numbers were determined by real-time PCR as described previously^3^ and normalized to nuclear DNA (28 s). Primer sequences utilized are shown in supplementary Table 1.

### Brown adipose tissue histology

Interscapular brown adipose tissues or liver tissue from all four groups of animals were embedded in paraffin, sectioned at 4 μm, and stained with hematoxylin and eosin-stain by standard method. Intracellular lipid content for brown adipose tissue was quantified by use of Nikon NIS-Elements BR software (NY, USA).

### Oil Red O staining of liver tissue

Fresh frozen liver tissues were cryostat sectioned at 6 μm and fixed in 4% PFA for 10 min at room temperature (RT). Slides were blotted in 60% isopropyl alcohol for 5 min, and then stained with filtered Oil Red O for 15 min at RT. Slides were rinsed once with distilled water, mounted, and cover slipped with warmed glycerol gelatin.

### Liver triglyceride measurement

For analysis of liver triglyceride (TG) content, approximately 50 mg of liver was placed into 500 μL of chilled Krebs Ringer Phosphate (118 mM NaCl, 5 mM KCl, 13.8 mM CaCl_2_, 1.2 mM MgSO_4_, 0.016% KH_2_PO_4_, 0.211% NaHCO_2_) and each sample was sonicated for ten times (30 seconds/time). The homogenate was centrifuged at 2,000 × *g* for 10 min at 4°C, and 10 μL of the supernatant was then removed for triglyceride analyses. Triglyceride content was measured using Wako triglyceride method kit (Richmond, USA).

### Immunohistochemical staining

Epididymal adipose tissue was fixed and embedded in paraffin. Paraffin-fixed adipose tissues were cut into 4 μm sections and placed onto slides. Sections were deparaffinized, rehydrated in graded mixtures of ethanol/water, pretreated by boiling in citrate buffer (pH 6.0), and endogenous peroxidase activity was blocked with 3% H_2_O_2_ for 30 min at room temperature (RT). The sections were incubated with a rat anti-mouse F4/80 antibody (AbD Serotec, Raleigh, NC) in blocking buffer for 1 hour at RT. Then, slides were washed, incubated with biotinylated secondary antibody for 30 min, and then washed again with PBS. Finally, peroxidase substrate diaminobenzidine (Vector Lab) was applied and incubated for 30 min. The slides were rinsed and counterstained with hematoxylin. Mounting solution and coverslips were added. Images were acquired with a Nikon Edipse 55i microscope.

### Macrophage migration assay

Bone-marrow derived cells were isolated from femurs and tibias of male WT and CD47 deficient mice fed with HF diet. These cells were cultured 7 days in RPMI-1640 media containing 20% FBS, 25 ng/ml M-CSF (Sigma), and penicillin/streptomycin to allow differentiation into mature macrophages. Ability of these macrophages to migrate toward MCP-1 (50 ng/ml) was determined using modified Boyden Microchemotaxis Chamber. Briefly, cells were washed twice with PBS, counted and loaded into the upper chambers of Transwell, while the lower chambers were filled with DMEM media with or without MCP-1 (50 ng/ml). Transwell plates were then incubated at 37°C for 6 hours. The upper inserts with membrane were removed and fixed in cold methanol and stained with crystal violet (Sigma). Cells were counted from five different fields for each well. Results were expressed as a migration index under the high magnification field.

### cGMP measurement

cGMP levels in brown fat or skeletal muscles from LF or HF fed WT or CD47-/- mice were measured by using the cGMP Direct Immunoassay Kit (Colorimetric) from Biovision. Frozen tissues were homogenized and the supernatant was collected. cGMP levels in the supernatant were measured and calculated based on the cGMP standard curve following the instruction manual.

### Western blotting analysis

Brow fat or skeletal muscle was homogenized in lysis buffer. After concentration being measured, homogenates were subjected to SDS-PAGE gel under reducing conditions and transferred onto a nitrocellulose membrane. After blocking, the membrane was incubated with anti-GAPDH (Millipore), anti-CD47 (BD Biosciences), or anti-PKG-I (BD Biosciences) antibodies at 4°C overnight. After washing, the membrane was incubated with horseradish peroxidase-conjugated secondary antibodies (Jackson Labs). The reaction was visualized by using an enhanced chemiluminescence system (Pierce). Immunoblots were analyzed by scanning densitometry and quantified by Quantity One gel Analysis software (Bio-Rad Laboratories).

### Statistical analysis

Data are expressed as the mean value ± SE. Statistical significance was assessed using ANOVA with Bonferroni ad hoc. A *p* value of <0.05 was considered statistically significant.

## Author Contributions

S.W. designed the experiments. H.M. and H.N. did the experiments in Figures 1–9. Z.Q. did the experiment in Figure 8 and 9. S.W. supervised the project. H.N. and S.W. wrote the manuscript. All authors reviewed the manuscript.

## Supplementary Material

Supplementary Information supplementary information

## References

[b1] Inoue M. *et al.* Thrombospondin 1 mediates high-fat diet-induced muscle fibrosis and insulin resistance in male mice. Endocrinology 154, 4548–4559, 10.1210/en.2013-1587 (2013). 24140711 PMC3836064

[b2] Varma V. *et al.* Thrombospondin-1 is an adipokine associated with obesity, adipose inflammation, and insulin resistance. Diabetes 57, 432–439 (2008). 18057090 10.2337/db07-0840 PMC2877915

[b3] Li Y., Tong X., Rumala C., Clemons K. & Wang S. Thrombospondin1 deficiency reduces obesity-associated inflammation and improves insulin sensitivity in a diet-induced obese mouse model. PLoS One 6, e26656 (2011). 22039525 10.1371/journal.pone.0026656 PMC3200349

[b4] Kong P. *et al.* Thrombospondin-1 regulates adiposity and metabolic dysfunction in diet-induced obesity enhancing adipose inflammation and stimulating adipocyte proliferation. Am J Physiol Endocrinol Metab 305, E439–450 (2013). 23757408 10.1152/ajpendo.00006.2013 PMC3742854

[b5] Mikhailenko I. *et al.* Cellular internalization and degradation of thrombospondin-1 is mediated by the amino-terminal heparin binding domain (HBD). High affinity interaction of dimeric HBD with the low density lipoprotein receptor-related protein. J Biol Chem 272, 6784–6791 (1997). 9045712 10.1074/jbc.272.10.6784

[b6] Goicoechea S., Orr A. W., Pallero M. A., Eggleton P. & Murphy-Ullrich J. E. Thrombospondin mediates focal adhesion disassembly through interactions with cell surface calreticulin. J Biol Chem 275, 36358–36368 (2000). 10964924 10.1074/jbc.M005951200

[b7] Chandrasekaran L. *et al.* Cell contact-dependent activation of alpha3beta1 integrin modulates endothelial cell responses to thrombospondin-1. Mol Biol Cell 11, 2885–2900 (2000). 10982388 10.1091/mbc.11.9.2885 PMC14963

[b8] Jimenez B. *et al.* Signals leading to apoptosis-dependent inhibition of neovascularization by thrombospondin-1. Nat Med 6, 41–48 (2000). 10613822 10.1038/71517

[b9] Frazier W. A. *et al.* The thrombospondin receptor integrin-associated protein (CD47) functionally couples to heterotrimeric Gi. J Biol Chem 274, 8554–8560 (1999). 10085089 10.1074/jbc.274.13.8554

[b10] Wang S. *et al.* Internalization but not binding of thrombospondin-1 to low density lipoprotein receptor-related protein-1 requires heparan sulfate proteoglycans. J Cell Biochem 91, 766–776 (2004). 14991768 10.1002/jcb.10781

[b11] Bornstein P. Thrombospondins as matricellular modulators of cell function. J Clin Invest 107, 929–934 (2001). 11306593 10.1172/JCI12749 PMC199563

[b12] Murphy-Ullrich J. E. & Mosher D. F. Interactions of thrombospondin with endothelial cells: receptor-mediated binding and degradation. J Cell Biol 105, 1603–1611 (1987). 2444599 10.1083/jcb.105.4.1603 PMC2114655

[b13] Taraboletti G., Roberts D. D. & Liotta L. A. Thrombospondin-induced tumor cell migration: haptotaxis and chemotaxis are mediated by different molecular domains. J Cell Biol 105, 2409–2415 (1987). 3680388 10.1083/jcb.105.5.2409 PMC2114831

[b14] Lawler J., Weinstein R. & Hynes R. O. Cell attachment to thrombospondin: the role of ARG-GLY-ASP, calcium, and integrin receptors. J Cell Biol 107, 2351–2361 (1988). 2848850 10.1083/jcb.107.6.2351 PMC2115659

[b15] Gao A. G. *et al.* Integrin-associated protein is a receptor for the C-terminal domain of thrombospondin. J Biol Chem 271, 21–24 (1996). 8550562 10.1074/jbc.271.1.21

[b16] Carlson C. B., Lawler J. & Mosher D. F. Structures of thrombospondins. Cell Mol Life Sci 65, 672–686 (2008). 18193164 10.1007/s00018-007-7484-1 PMC2578829

[b17] Matozaki T., Murata Y., Okazawa H. & Ohnishi H. Functions and molecular mechanisms of the CD47-SIRPalpha signalling pathway. Trends Cell Biol 19, 72–80, 10.1016/j.tcb.2008.12.001 (2009). 19144521

[b18] Oldenborg P. A. CD47: A Cell Surface Glycoprotein Which Regulates Multiple Functions of Hematopoietic Cells in Health and Disease. ISRN Hematol 2013, 614619, 10.1155/2013/614619 (2013). 23401787 PMC3564380

[b19] Brown E. J. & Frazier W. A. Integrin-associated protein (CD47) and its ligands. Trends Cell Biol 11, 130–135 (2001). 11306274 10.1016/s0962-8924(00)01906-1

[b20] Barclay A. N. & Van den Berg T. K. The Interaction Between Signal Regulatory Protein Alpha (SIRPalpha) and CD47: Structure, Function, and Therapeutic Target. Annu Rev Immunol 32, 25–50, 10.1146/annurev-immunol-032713-120142 (2014). 24215318

[b21] Rogers N. M. *et al.* Activated CD47 regulates multiple vascular and stress responses: implications for acute kidney injury and its management. Am J Physiol Renal Physiol 303, F1117–1125, 10.1152/ajprenal.00359.2012 (2012). 22874763 PMC3469673

[b22] Isenberg J. S. *et al.* Thrombospondin-1 and CD47 regulate blood pressure and cardiac responses to vasoactive stress. Matrix Biol 28, 110–119, 10.1016/j.matbio.2009.01.002 (2009). 19284971 PMC2663008

[b23] Isenberg J. S., Shiva S. & Gladwin M. Thrombospondin-1-CD47 blockade and exogenous nitrite enhance ischemic tissue survival, blood flow and angiogenesis via coupled NO-cGMP pathway activation. Nitric Oxide 21, 52–62 (2009). 19481167 10.1016/j.niox.2009.05.005 PMC2768066

[b24] Isenberg J. S., Frazier W. A., Krishna M. C., Wink D. A. & Roberts D. D. Enhancing cardiovascular dynamics by inhibition of thrombospondin-1/CD47 signaling. Curr Drug Targets 9, 833–841 (2008). 18855617 10.2174/138945008785909338 PMC2575641

[b25] Isenberg J. S. *et al.* CD47 is necessary for inhibition of nitric oxide-stimulated vascular cell responses by thrombospondin-1. J Biol Chem 281, 26069–26080 (2006). 16835222 10.1074/jbc.M605040200

[b26] Yao M., Roberts D. D. & Isenberg J. S. Thrombospondin-1 inhibition of vascular smooth muscle cell responses occurs via modulation of both cAMP and cGMP. Pharmacol Res 63, 13–22, 10.1016/j.phrs.2010.10.014 (2011). 20971192 PMC3026097

[b27] Ramanathan S., Mazzalupo S., Boitano S. & Montfort W. R. Thrombospondin-1 and angiotensin II inhibit soluble guanylyl cyclase through an increase in intracellular calcium concentration. Biochemistry 50, 7787–7799, 10.1021/bi201060c (2011). 21823650 PMC3168727

[b28] Wajchenberg B. L. Subcutaneous and visceral adipose tissue: their relation to the metabolic syndrome. Endocr Rev 21, 697–738, 10.1210/edrv.21.6.0415 (2000). 11133069

[b29] Weisberg S. P. *et al.* Obesity is associated with macrophage accumulation in adipose tissue. J Clin Invest 112, 1796–1808 (2003). 14679176 10.1172/JCI19246 PMC296995

[b30] Koppaka S. *et al.* Reduced adipose tissue macrophage content is associated with improved insulin sensitivity in thiazolidinedione-treated diabetic humans. Diabetes 62, 1843–1854 (2013). 23349486 10.2337/db12-0868 PMC3661618

[b31] Xu H. *et al.* Chronic inflammation in fat plays a crucial role in the development of obesity-related insulin resistance. J Clin Invest 112, 1821–1830 (2003). 14679177 10.1172/JCI19451 PMC296998

[b32] Frazier E. P. *et al.* Age-dependent regulation of skeletal muscle mitochondria by the thrombospondin-1 receptor CD47. Matrix Biol 30, 154–161, 10.1016/j.matbio.2010.12.004 (2011). 21256215 PMC3070423

[b33] Cannon B. & Nedergaard J. Brown adipose tissue: function and physiological significance. Physiol Rev 84, 277–359 (2004). 14715917 10.1152/physrev.00015.2003

[b34] Cannon B. & Nedergaard J. Metabolic consequences of the presence or absence of the thermogenic capacity of brown adipose tissue in mice (and probably in humans). Int J Obes (Lond) 34 Suppl 1, S7–16, 10.1038/ijo.2010.177 (2010). 20935668

[b35] Richard D. & Picard F. Brown fat biology and thermogenesis. Frontiers in bioscience (Landmark edition) 16, 1233–1260 (2011). 21196229 10.2741/3786

[b36] Schreurs M., Kuipers F. & van der Leij F. R. Regulatory enzymes of mitochondrial beta-oxidation as targets for treatment of the metabolic syndrome. Obesity reviews: an official journal of the International Association for the Study of Obesity 11, 380–388, 10.1111/j.1467-789X.2009.00642.x (2010). 19694967

[b37] Handa P. *et al.* Reduced vascular nitric oxide-cGMP signaling contributes to adipose tissue inflammation during high-fat feeding. Arterioscler Thromb Vasc Biol 31, 2827–2835, 10.1161/atvbaha.111.236554 (2011). 21903940 PMC3342311

[b38] Rizzo N. O. *et al.* Reduced NO-cGMP signaling contributes to vascular inflammation and insulin resistance induced by high-fat feeding. Arterioscler Thromb Vasc Biol 30, 758–765, 10.1161/atvbaha.109.199893 (2010). 20093624 PMC2865555

[b39] Sick E. *et al.* CD47 update: a multifaceted actor in the tumour microenvironment of potential therapeutic interest. Br J Pharmacol 167, 1415–1430, 10.1111/j.1476-5381.2012.02099.x (2012). 22774848 PMC3514757

[b40] Willingham S. B. *et al.* The CD47-signal regulatory protein alpha (SIRPa) interaction is a therapeutic target for human solid tumors. Proc Natl Acad Sci U S A 109, 6662–6667, 10.1073/pnas.1121623109 (2012). 22451913 PMC3340046

[b41] Soto-Pantoja D. R., Isenberg J. S. & Roberts D. D. Therapeutic Targeting of CD47 to Modulate Tissue Responses to Ischemia and Radiation. J Genet Syndr Gene Ther 2 (2011). 10.4172/2157-7412.1000105 PMC3368437 22685691

[b42] Maxhimer J. B., Shih H. B., Isenberg J. S., Miller T. W. & Roberts D. D. Thrombospondin-1/CD47 blockade following ischemia-reperfusion injury is tissue protective. Plast Reconstr Surg 124, 1880–1889, 10.1097/PRS.0b013e3181bceec3 (2009). 19952644 PMC2794041

[b43] Kanda H. *et al.* MCP-1 contributes to macrophage infiltration into adipose tissue, insulin resistance, and hepatic steatosis in obesity. J Clin Invest 116, 1494–1505 (2006). 16691291 10.1172/JCI26498 PMC1459069

[b44] Weisberg S. P. *et al.* CCR2 modulates inflammatory and metabolic effects of high-fat feeding. J Clin Invest 116, 115–124 (2006). 16341265 10.1172/JCI24335 PMC1307559

[b45] Kamei N. *et al.* Overexpression of monocyte chemoattractant protein-1 in adipose tissues causes macrophage recruitment and insulin resistance. J Biol Chem 281, 26602–26614 (2006). 16809344 10.1074/jbc.M601284200

[b46] Sullivan T. J. *et al.* Experimental evidence for the use of CCR2 antagonists in the treatment of type 2 diabetes. Metabolism 62, 1623–1632 (2013). 23953944 10.1016/j.metabol.2013.06.008

[b47] Gutierrez D. A. *et al.* Aberrant accumulation of undifferentiated myeloid cells in the adipose tissue of CCR2-deficient mice delays improvements in insulin sensitivity. Diabetes 60, 2820–2829 (2011). 21926275 10.2337/db11-0314 PMC3198070

[b48] Oh D. Y., Morinaga H., Talukdar S., Bae E. J. & Olefsky J. M. Increased macrophage migration into adipose tissue in obese mice. Diabetes 61, 346–354 (2012). 22190646 10.2337/db11-0860 PMC3266418

[b49] Haas B. *et al.* Protein kinase G controls brown fat cell differentiation and mitochondrial biogenesis. Science signaling 2, ra78, 10.1126/scisignal.2000511 (2009). 19952371

[b50] Mitschke M. M. *et al.* Increased cGMP promotes healthy expansion and browning of white adipose tissue. Faseb j 27, 1621–1630, 10.1096/fj.12-221580 (2013). 23303211

[b51] Nikolic D. M., Li Y., Liu S. & Wang S. Overexpression of constitutively active PKG-I protects female, but not male mice from diet-induced obesity. Obesity (Silver Spring) 19, 784–791, 10.1038/oby.2010.223 (2011). 20930715 PMC9125568

