# Supplementary material for: CD47 Deficiency Protects Mice From Diet-induced Obesity and Improves Whole Body Glucose Tolerance and Insulin Sensitivity
Source: Sci Rep. 2015 Mar 9;5:8846. doi: 10.1038/srep08846 (PMC4352923; doi:10.1038/srep08846)

CD47 Deficiency Protects Mice From Diet-induced Obesity and Improves Whole Body Glucose Tolerance and Insulin Sensitivity

Hasiyeti Maimaitiyiming[1], Heather Norman[1], Qi Zhou and Shuxia Wang[*]

Department of Pharmacology and Nutritional Sciences, University of Kentucky, Lexington, KY 40536; Lexington Veterans Affairs Medical Center, Lexington, KY

[1] These authors contribute equally to the work.

Supplementary Figure: Full-length western blot images for Figures 8A, 9B and 9D.
A). Full-length western blots to show CD47 protein levels in brown adipose tissue from LF or HF fed WT mice; B). Full-length western blots to show PKG-I protein levels in brown adipose tissue from LF or HF fed WT mice; and C). Full-length western blots to show PKG-I protein levels in skeletal muscle from LF or HF fed WT mice by immunoblotting.

A). Full-length blots of CD47 in brown fat

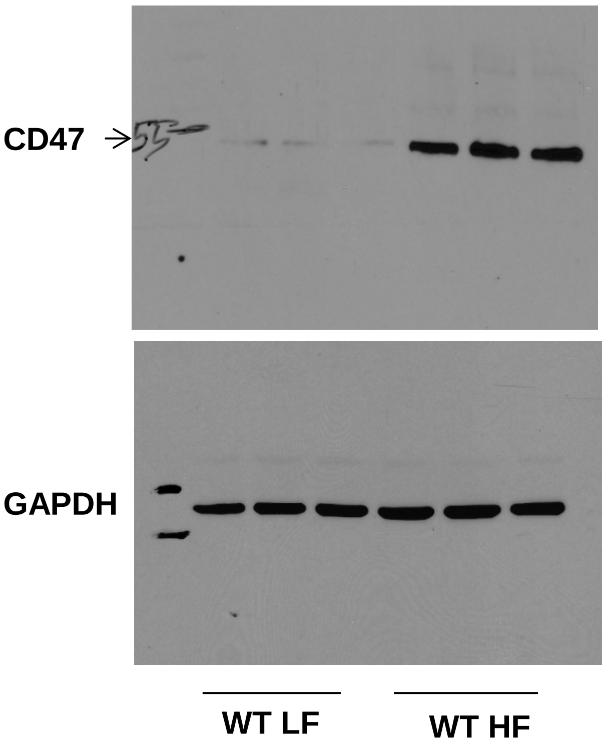

B). Full-length blots of PKG-1 in brown fat

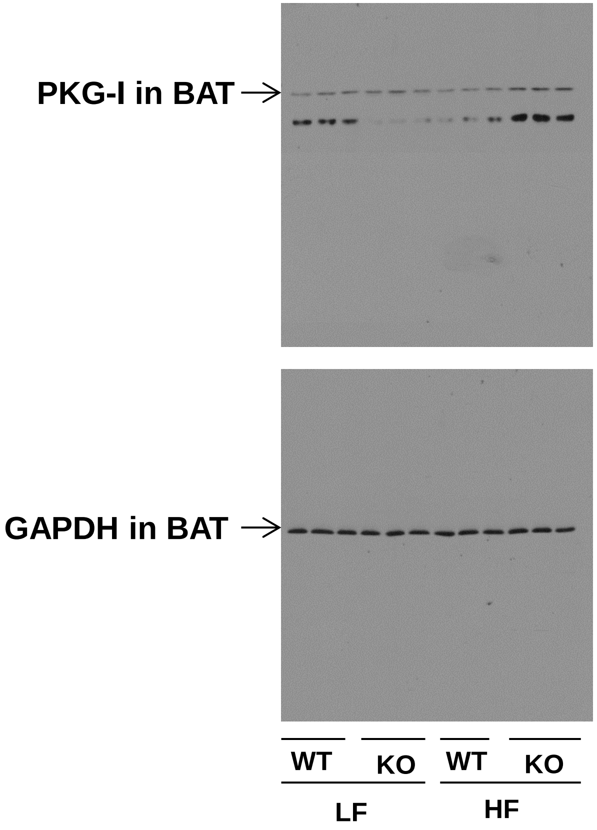

C). Full-length blots of PKG-1 in muscle

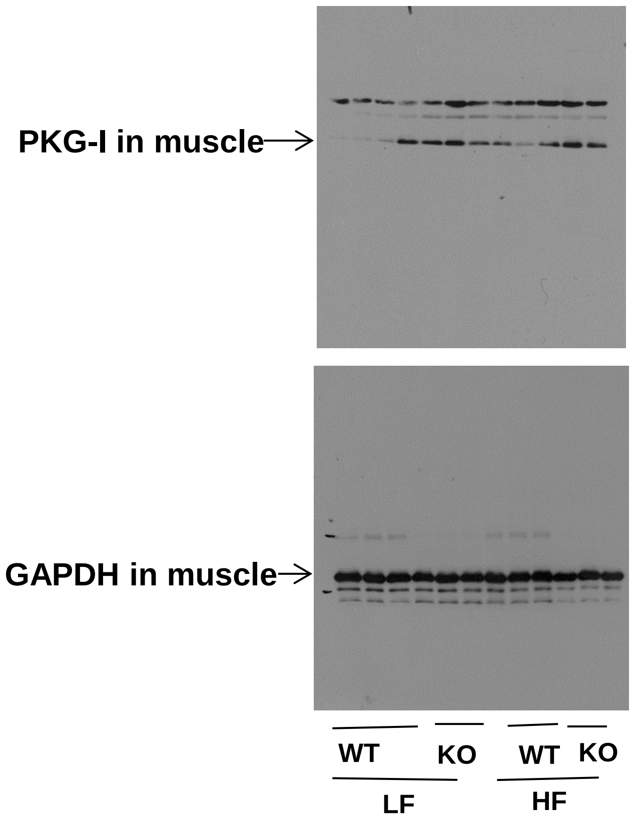

Supplement: Supplementary Information [file srep08846-s1.pdf]
